# Evaluating the applicability of MESS (matrix exponential spatial specification) model to assess water quality using GIS technique in agricultural mountain catchment (Western Carpathian)

**DOI:** 10.1007/s10661-018-7137-x

**Published:** 2018-12-21

**Authors:** Wiktor Halecki, Tomasz Stachura, Wioletta Fudała, Maria Rusnak

**Affiliations:** 1 Department of Land Reclamation and Environmental Development, Faculty of Environmental Engineering and Land Surveying, University of Agriculture in Krakow, Al. Mickiewicza 24-28, 30-059 Kraków, Poland; 2 University of Agriculture in Krakow, Kraków, Poland

**Keywords:** Flysch slope, GIS techniques, Land use, Multivariate analysis, Water quality indices

## Abstract

The formation of many sources of pollution in a short period of time is due to mountain soil erosion by water. One of the major mechanisms decisive in the intensification of such erosion is the loosening of soil material on the slope. Water quality studies show the impact of diversified spatial management and allow making the right decisions in environmental management in mountain areas with high variability of use and land cover. The research undertaken as part of the paper was carried out in order to determine the dependency between total suspended solids (TSS) and the physicochemical parameters of surface waters and the amount of soil losses in the use structure within the mountain catchment. The paper focused on the frequency of phenomena in time and the possibility of stopping the surface runoff on the slope and on the soil’s susceptibility to water erosion. The dependencies between multipoint sampling and the concentration of material washed off the slope due to precipitation were verified with a multivariate analysis. Sampling took place in hydrometric sections, and during small floods, in the waterbed cross section. Research shows that such sampling is the basis for the calculation of the transported load, reflecting the average variation in concentration. The variation in the volume of the load from the individual parts of the catchment was assessed by the spatial autoregressive model. It was found that the use of river basin areas affects water chemistry. Water reservoirs are an important ecological barrier for the migration of nitrate nitrogen (N-NO_3_) and phosphate phosphorus (P-PO_4_), which is marked by changes in the growing season. Water along the sections of the river near the quarry with a high degree of sodding showed good quality condition. Despite significant differences between measurement sampling sites, high total dissolved solid (TDS) values were found in communities adjacent to forests and meadows. However, the highest electrical conductivity (EC) and TSS concentrations were found in the interface with cultivated areas. Biogenic indices showed variation depending on the way the adjacent areas were used. GIS linked spatial variables with the formation of water pollution. The analysis of spatial autoregression pointed to the impact of arable land. Moreover, the analysis of spatial autoregression with the MESS function designated a connection between agricultural land use and nitrite nitrogen (N-NO_2_), EC, TSS, and dissolved oxygen (DO).

**Electronic supplementary material:**

The online version of this article (10.1007/s10661-018-7137-x) contains supplementary material, which is available to authorized users.

**Graphical abstract:**
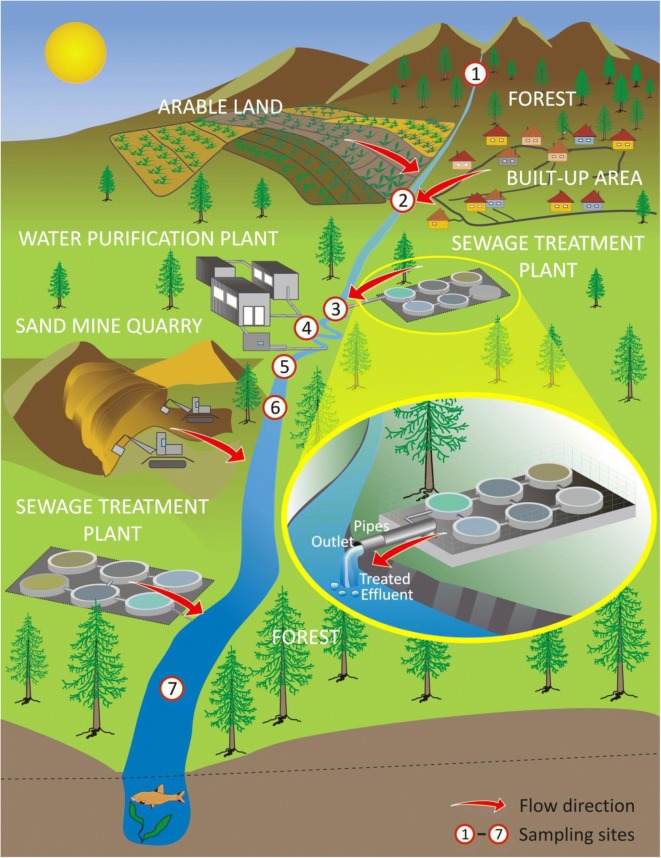
ᅟ

## Introduction

Water pollution occurs at all stages of its circulation in the biosphere. The main sources of pollution are as follows: municipal and industrial sewage, mining water, cooling water from the power industry (Bugajski et al. 2016a; Liao et al. 2016), and runoff from agricultural areas (Priess et al. 2015). The river transports various surface water pollutants. Monitoring for the purpose of drinking water supply for the population at various control and measurement points should include nitrate nitrogen and ammonium nitrogen in assessing the level of pollution (Cheung et al. 2003; Bharti and Katyal 2011; Barbaruah et al. 2012; Gu et al. 2014; Jasmin and Mallikarjuna 2014; Dash et al. 2015). Water usability is the primary criterion in the scale of surface water quality assessments (Terrado et al. 2010; Ahmed et al. 2014; Lychagin et al. 2015; Dąbrowska et al. 2017). The quality assessment is based on the value of several dozen physical, chemical, and biological indices (Bhutiani et al. 2014). The first group of indices is as follows: color, temperature, odor, turbidity, electrical conductivity, and pH. The second group of indices enables assessment of oxygen balance in water. The third group of indices allows determining the content of, e.g., organic carbon, organic nitrogen, heavy metals, detergents, phosphates, chlorides, sulfates, and artificial fertilizers. Indices of the last group are related to sanitary and epidemiological parameters. They include tests for pathogenic and fecal bacteria as well as the presence of various indicator organisms (Rai et al. 2010; Luis et al. 2011; Kim et al. 2012; Alves et al. 2014; Hayzoun et al. 2015). There is a particularly strong relationship between the size of the slurry fraction and the concentration of heavy metals adsorbed (Fu et al. 2014; Dai et al. 2015). The presence of phosphates in water is the result of natural washout processes from minerals and rocks, penetration of phosphorus compounds used in soil management and plant protection (organophosphorus insecticides), and urban or industrial sewage pollution (Svanbäck et al. 2014). The content of ammonia itself is not essential in the water’s assessment in terms of hygienic and sanitary aspects. It is an indicator of contamination of economic wastewater, where pathogenic bacteria may be present (Moore and Langner 2012). Without the accompanying nitrites and nitrates, the presence of ammonia itself in the water shows nearby and quite recent water pollution (Policht-Latawiec and Żarnowiec 2017). Organic and mineral fertilization promotes water contamination with nitrates (Żarnowiec et al. 2017). The concentration of solutes greatly affects oxygen’s water solubility, and thus the ability of water to assimilate impurities. The abundance of alkaline ions indicates their origin from rocks (Kyllmar et al. 2014) or in soils (Fernández et al., 2012; Buelow et al. 2015; Ulén and Snäll 2007; Ulén and Etana 2014). The power source indicates a supply of fresh material from surrounding areas. The primary ions indicate the alkalinity of water (Oster et al. 2016). Sulfates formed during organic decay are then evidence of water contamination. The increased content of chloride ions in river water comes from wastewater discharges. Municipal sewage, especially fecal, shows a high content of chlorides. Source of sodium and potassium in surface water is pollution from industrial wastewater, especially one coming from soda factories, processing of mineral salts, and production of sodium and potassium. Potassium ions reach natural waters from fields fertilized with potassium salts and from animal and plant wastes (Smith et al. 2015). Calcium and magnesium reach natural waters from the soil and leaching dolomites, magnesite, phosphogypsum, and other minerals containing calcium and magnesium compounds (Vyshpolsky et al. 2010; Potasznik and Szymczyk 2015). Source of iron compounds in natural water is sewage and industrial waste as well as corrosion of pipelines, tanks, equipment, and iron structures. Significant amounts of iron are found in mining waters and in sewage from ore refinement plants, etching plants, or chemical plants (Ziadat et al. 2015). Manganese usually occurs in natural water together with iron. It penetrates into groundwater as a result of corrosion of metals, from plant residues and sewage, mainly industrial, such as metallurgical, electrotechnical, and chemical industries (Parmar and Keshari 2012; Bhat et al. 2014; Wąsik and Chmielowski 2016). Checking the limits of their concentration is important to assess the degradation of surface water (Juahir et al. 2010; Weber et al. 2014; Ahmed et al. 2015; Assouline et al. 2015; Hu et al. 2015; Bugajski et al. 2016b).

Surface runoff on arable lands and strongly developed groove erosion (soil particles joining into longitudinal streams under precipitation) on the surface of pastures degrade the soil on the flysch slope (Halecki et al. 2017). The assessment of physicochemical indices in quantitative research is therefore correct, because these parameters are the carrier of hydrological characteristics of water and indirect proof of the occurrence of surface (rill) erosion (Stallard and Murphy 2014). This may be evidence of deteriorating water flow conditions and occurrence of a hardly permeable or smaller surface runoff due to lower total suspended solids. Based on that, research hypotheses and the plan of the whole paper were put forward. Particularly important dependencies between land use on the mountain slope and the physicochemical parameters of the mountain catchment were verified using the following research hypotheses: (i) there is a relationship between land use and physicochemical indices of surface water; (ii) the source of suspended sediment (total suspended solids) in surface water are arable areas on the mountain slope.

## Material and methods

### Research area

**Fig. 1:**
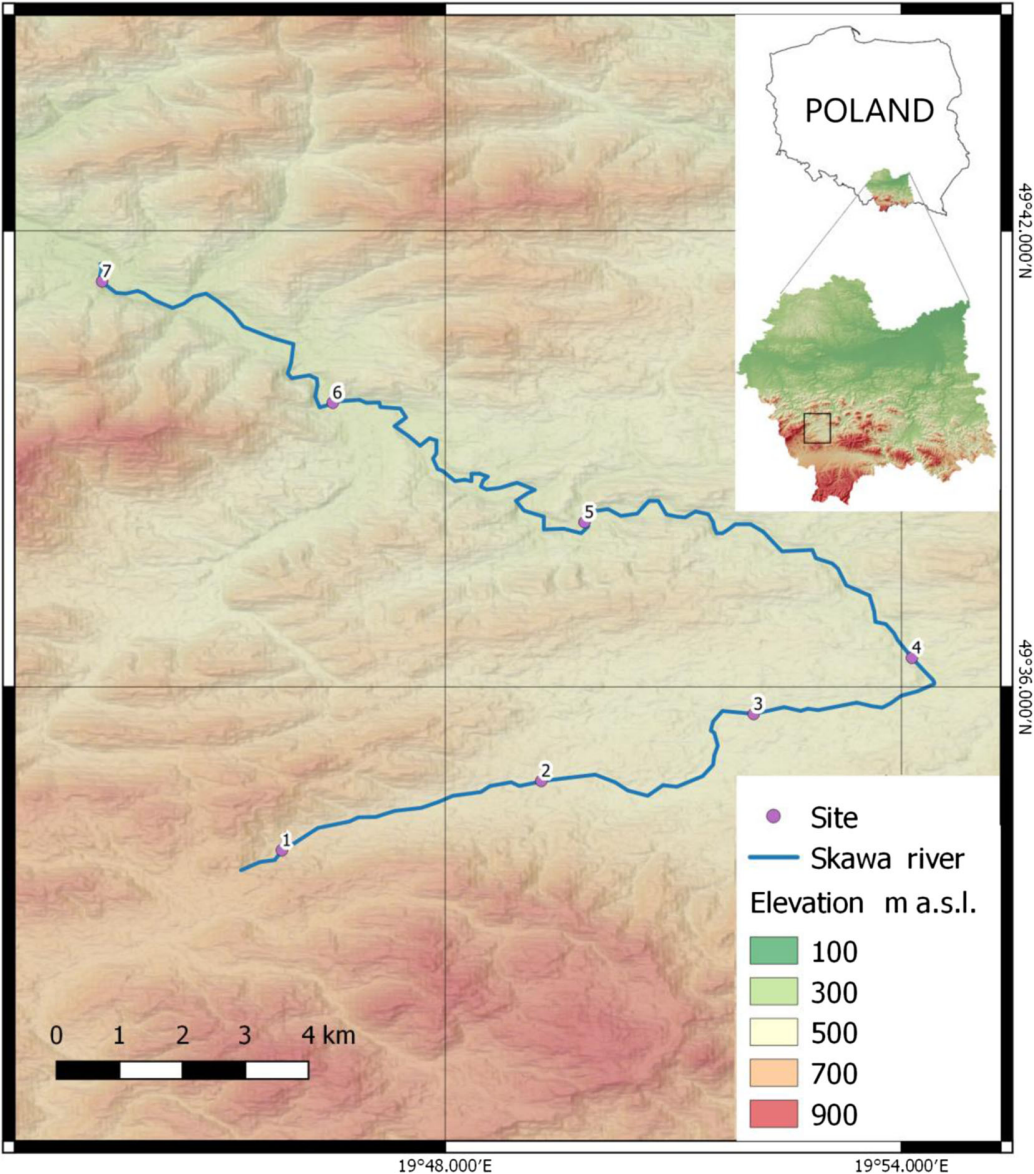
Location of the research area with relief

The research area was located in the south-western part of the Małopolska (Lesser Poland) province (Fig. 1) in Poland. The valleys of streams are filled with material from river-glacial accumulation. These are primarily stone-gravel deposits covered with a layer of clay carried by water from mountain slopes. The area is made of flysch formations of the Magura nappe, covered with a continuous coat of Quaternary layers. Flysch formations are formed as sandstones with subordinate interbeddings of clay and marly shales. The highest elevation of the water section is 864.9 m above sea level. The Skawa river basin in the area is typically mountainous, characterized by high variability of flows and a high incidence of floods, especially in the summer. The quality of water in the Skawa river was determined on the basis of seven series of water sample analyses taken at seven measurement points in the Spytkowice and Jordanów municipalities. The river Skawa, which is the subject of research, borders with the Soła catchment on the west, with the Raba catchment on the east, and on the south with the Orawa catchment, belonging to the Dunajec basin. Furthermore, the watershed of the Skawa river is partly a state border with Slovakia. The density of the hydrographic network is 1.8 km^−1^ km^−2^ and is a high value for the source section of the Carpathian tributaries of the Vistula.

### Sampling area

**Fig. 2:**
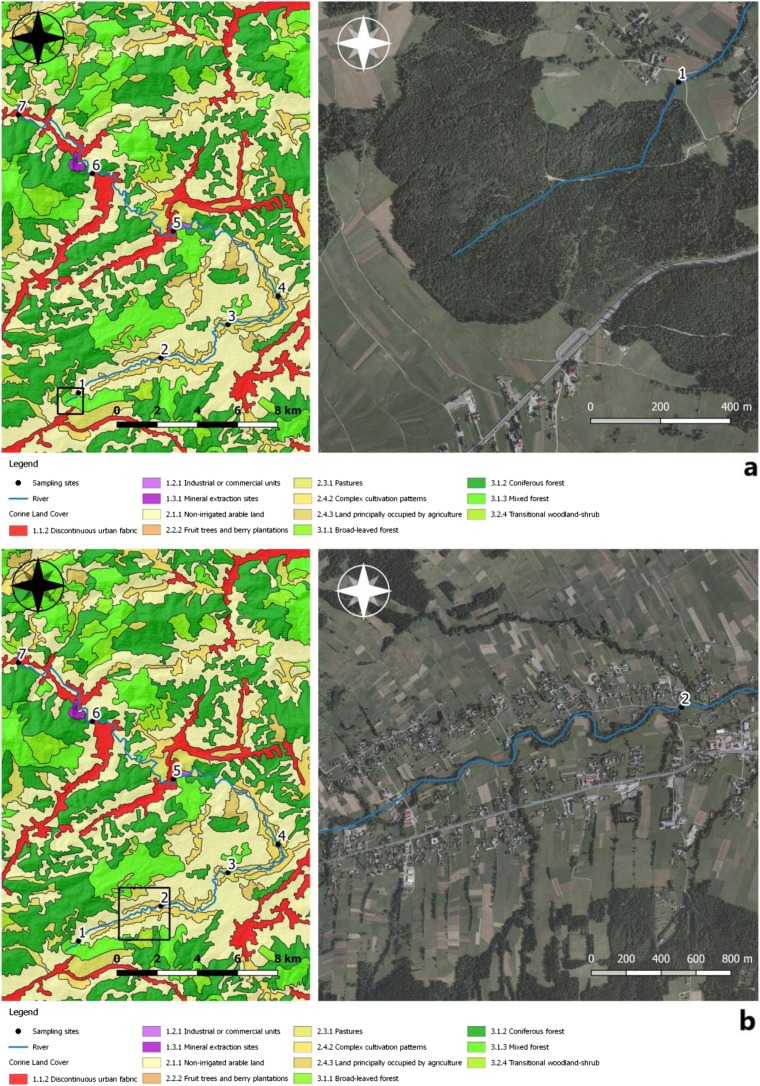

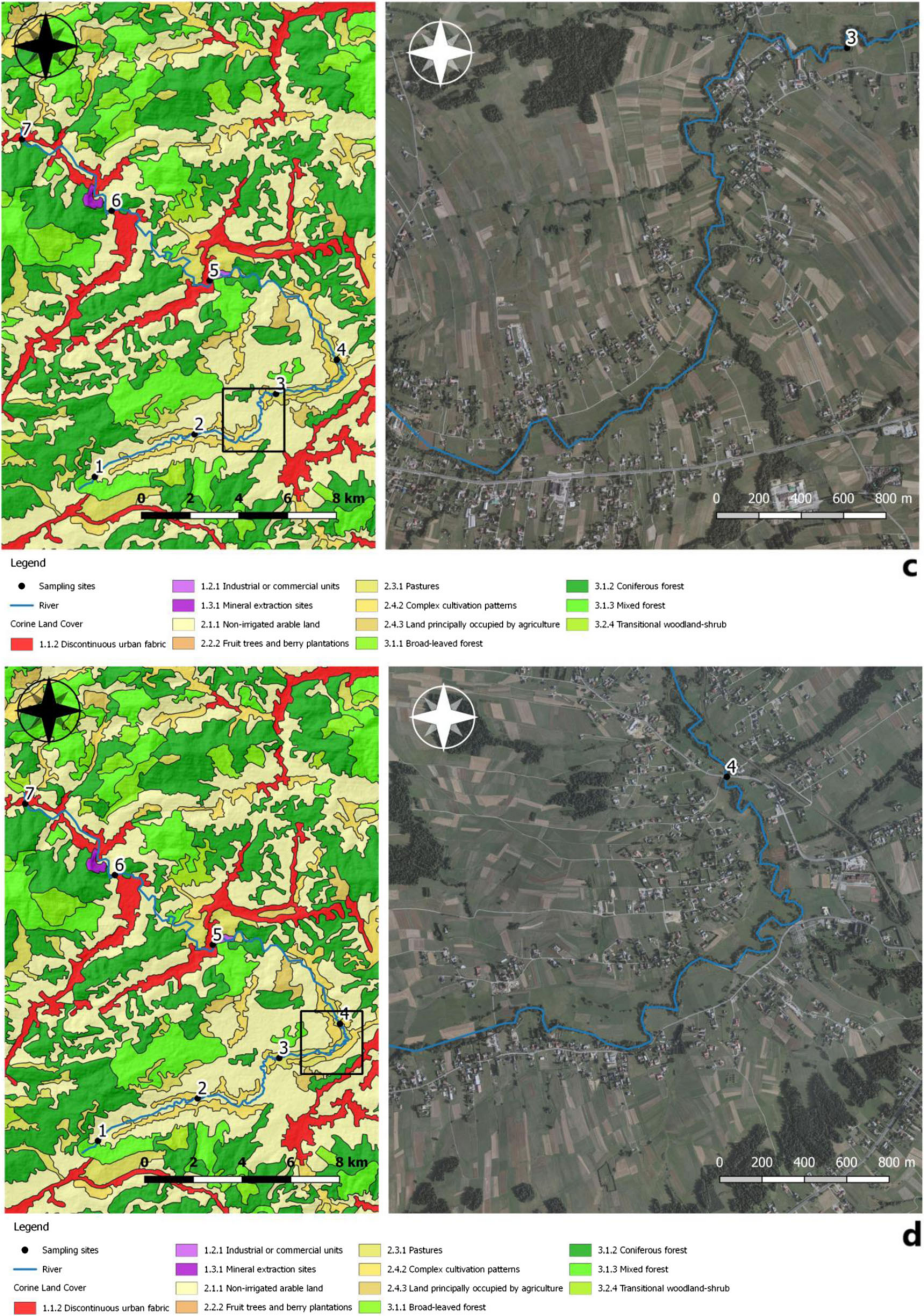

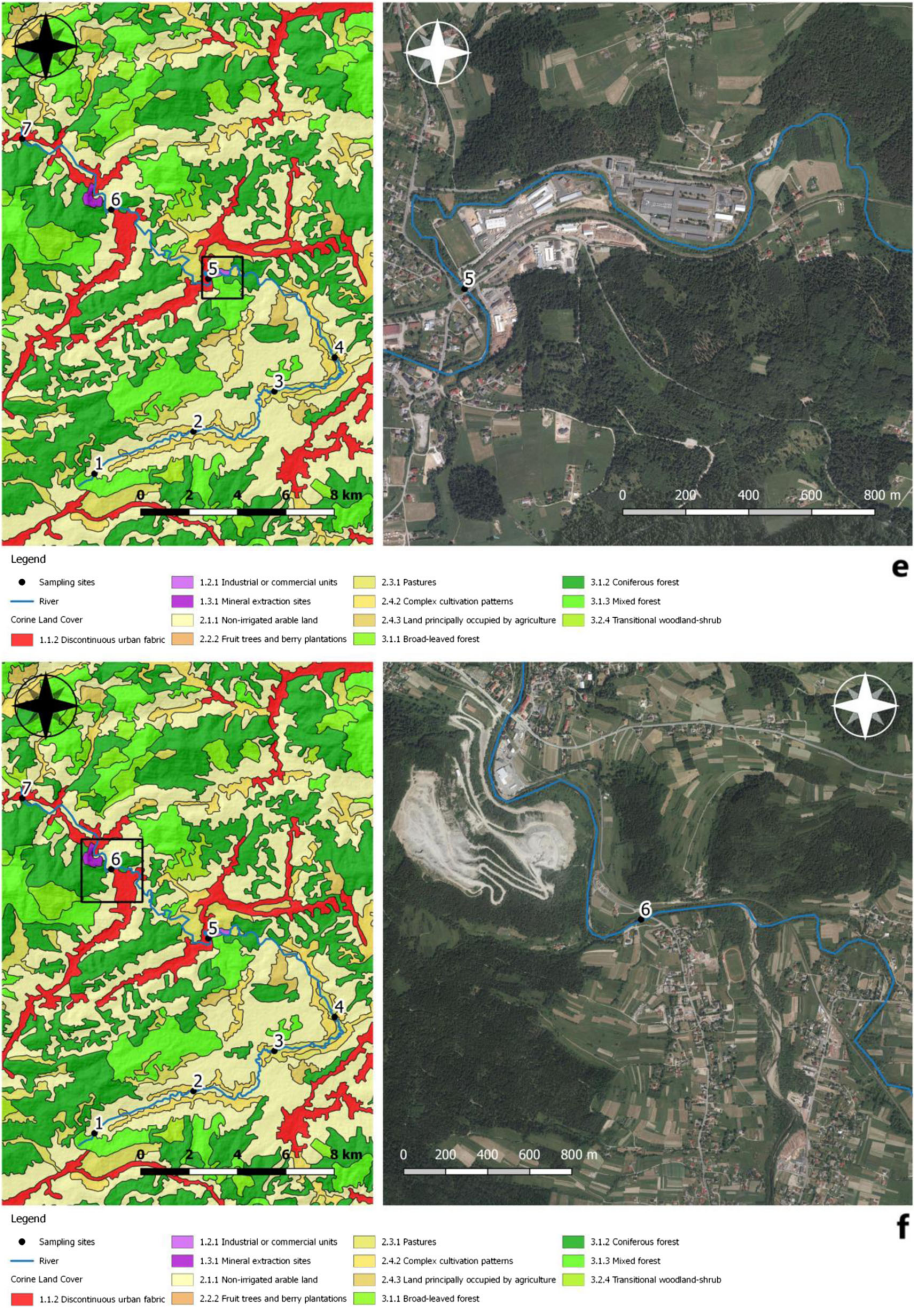

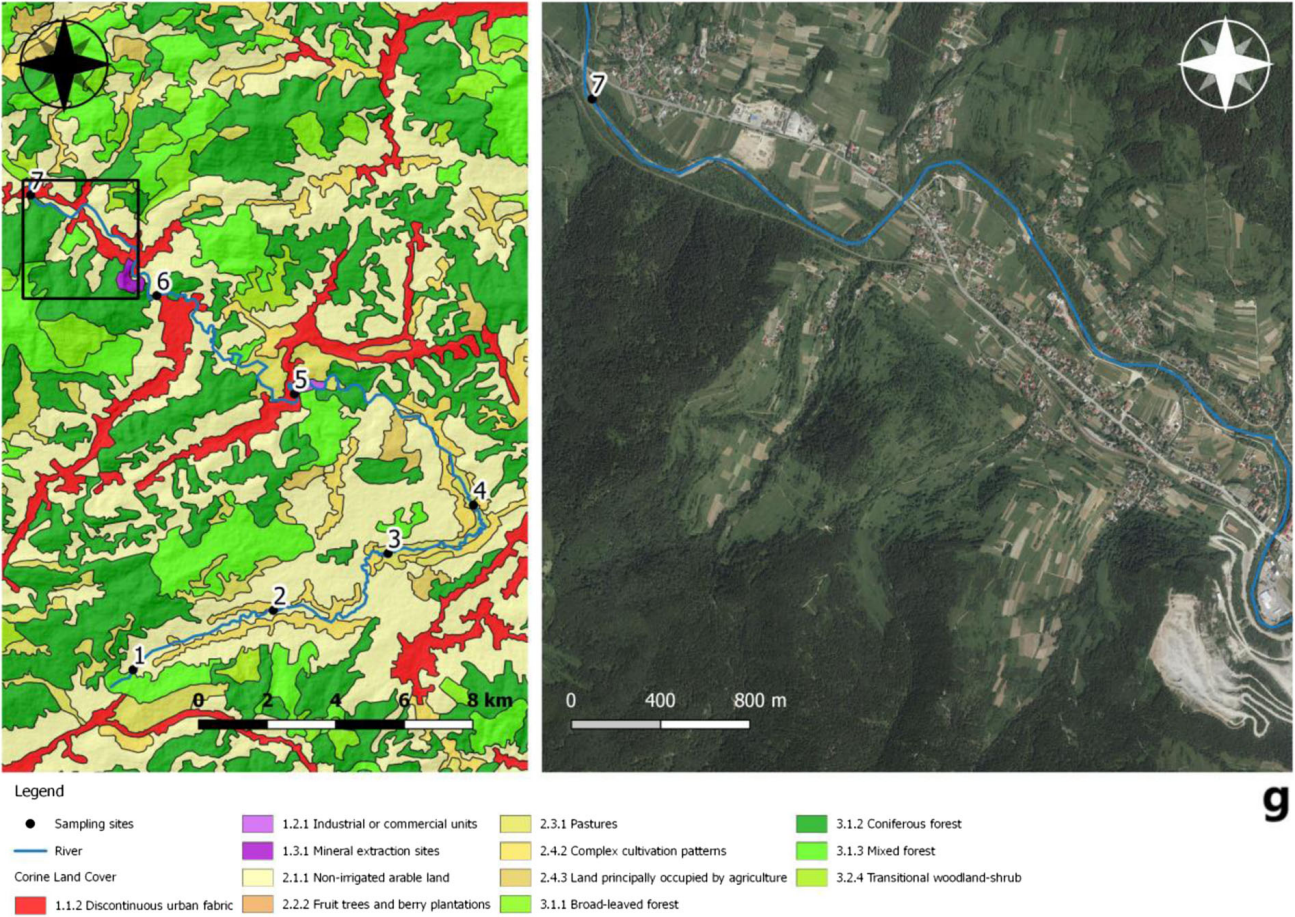
**a**–**g** Sampling locations and land use. The direction of the mountain watercourse has been marked on orthophomaps

Sampling sites were most often located at the interface of the Skawa river with technical infrastructure, mainly on bridges (Fig. 2a–g). These locations were chosen due to anthropogenic pollution there. A total of 28 samples were collected from seven sites after each field visit. The first sampling site was located at an altitude of approx. 622 m above sea level, at a distance of 852 m from the source of the Skawa river. These are forest areas with anthropogenic impact present. The second sampling site was located at 524 m above sea level; on the section with a length of 5.64 km, the surrounding areas were used for agricultural purposes, with relatively dense buildings and an incomplete sewage system. The river was fed with pollution from households and surface runoff from fields and roads. The third sampling site was located at an altitude of approx. 482 m above sea level. The length of the stretch of watercourse was 6.29 km. It was chosen due to close proximity to sewage treatment plants, a slaughterhouse and a tannery. The fourth sampling site was selected at an altitude of 471 m. The section was about 4 km long, and the river in this place had a strong meandering aspect. The fifth sampling site was at an altitude of 447 m just behind the water intake located in the upper section of the river Skawa (1500 m from the railway bridge located at the Jordanów railway station). The waterworks consisted of the following facilities: a double surface water intake, a water treatment station, a water main, and a distribution water supply network, along with network reservoirs. The sixth sampling site was located at an altitude of 421 m above sea level, in front of the “OSIELEC” sandstone mine. It is a monumental, several-level, and operational quarry exploiting Magura sandstone. This excavation stretches from the ravine section of Skawa up to the very top of the mountain. It is the largest sandstone deposit in Lesser Poland and one of the largest in Poland: over 65 million tons of raw materials. There is a municipal mechanical and biological sewage treatment plant type SBR between the measurement/control points 6 and 7 in Osielec, with a sequential batch reactor. The treatment plant is based on the low-load activate sludge method, with simultaneous oxygen stabilization of excess sludge. The mountain aspect of the Skawa hydrological regime makes it a river with low hydrological inertia. Therefore, it is characterized by a significant amplitude of flow variability. Skawa is a river with a significant flood potential; it is characterized by violent but short-lived floodings. The seventh sampling site was chosen at 387 m above sea level, and it was near a railway station surrounded by forests.

### Determination of physicochemical parameters

The parameters tested directly in the field were as follows:

- pH of water by potentiometric method using a CP-104 type pH meter

- Electrical conductivity (EC) of the water expressed in μS cm^−1^ using the conductometric method with an Elmetron CC-101 conductometer

- Dissolved oxygen (DO) in content in mg dm^−3^ and degree of water saturation with oxygen in % using the electrochemical method with an Elmetron CO-411 oxygen meter

- Water temperature expressed in °C with a digital thermometer embedded in a water-tight CO-411 oxygen meter

- Total dissolved sediments (TDS) in mg dm^−3^ using HM digital meter

### Determination of nutrients and trace elements

The determinations were made using reference methods (Regulation of the Minister of the Environment 2016) to establish values and concentrations of water quality indices. Calcium (Ca^2+^), sodium (Na^+^), potassium (K^+^), magnesium (Mg^2+^), total iron (Fe_tot_), and manganese (Mn^2+^) were determined using atomic absorption spectrometry on a Unicam Solar 969 apparatus. Ammonium nitrogen (N-NH_4_^+^), nitrate nitrogen (N-NO_3_^−^), nitrite nitrogen (N-NO_2_^−^), phosphate phosphorus (P-PO_4_), and chlorides (Cl^−^) were determined using a flow (injection) colorimetric analysis on a computer-controlled FIAstar 5000 camera. Sulfates (SO_4_^2−^) and total suspended solids (TSS) were established using the gravimetric method. The samples were collected using the bathymetric method, and the concentration of the substance was calculated using a filtration technique and the mass of material filtered on laboratory filters. Biochemical oxygen demand was defined with the Winkler method. Chemical oxygen demand was obtained with the titration method.

### Spatial model and statistical analysis

In the study of spatial dependencies, we used a modification for the spatial autoregressive structure and performed calculations using a matrix exponential spatial specification (MESS) (LeSage and Parent 2007). In our system (MESS), the explained variable was replaced by a transformation, which was determined by the exponential matrix of the analysis for physicochemical indices related to the neighborhood matrix:

$$ S={e}^{\alpha W}={\sum}_{i=0}^{\infty }\ \frac{\alpha^i{W}^i}{i!} $$

*S*: Exponential matrix of dependent variable for the spatial model
*α*: Strength of spatial autocorrelation for *i-*parameter of explained variable
*W*: Neighborhood matrix for the observed variable

The MESS spatial model is defined as:

$$ {e}^{\alpha W}Y= X\beta +\varepsilon $$

where

*e*: Exponential function for dependent variable *Y*
*Xβ*: Matrix of observations for vector of regression coefficient
*ε*: Value of the random component (observation error)

In this article, the model has been generalized to include spatial dependencies in the entire research area using the model’s autocovariance function. Various types of land use were spatially divided between sampling sites to average the neighbors’ impact (in the neighborhood matrix) on the observation. The SAR (simultaneous autoregressive) is a special case of the model, in which the dependent variable is subject to linear transformation. SAR model is given by the formula:

$$ Y=\rho Wy+ X\beta +\varepsilon $$

where

*Y*: Dependent variable for the spatial model
*y*: Value of lagged dependent variable (delayed) in time (explicative variables at site between measurements)
*ρ*: Spatial effect coefficient (autocorrelation parameter)
*Wy*: Neighborhood matrix for the observed variable *y* (the elements of a row-standardized matrix of spatial weights)
*Xβ*: Matrix of observations for vector of regression coefficient
*ε*: Value of the random component (observation error)

The spatial autoregression parameter reflects the strength of the relation between observations of the variable explained in different locations. This innovation greatly facilitates the estimation of model parameters in comparison with the traditional spatial approach. Classical linear regression model and spatial autoregression were performed with SAM (Spatial Analysis in Macroecology) software, version 4.0 (Rangel et al. 2010).

This article also defines the descriptive statistics of the parameters, i.e., the minimum and maximum values, the arithmetic mean, the center value (median), and the coefficients of parameter variation at individual measurement-control points. The variability of selected indices in the examined points during the research period is presented. Statistical conclusions regarding the significance of differences in the values of indices between measurement-control points were carried out using the Kruskal–Wallis test. A non-parametric test was chosen due to the lack of distribution normality of the analyzed parameters in each analyzed measurement-control point, in accordance with the results of the Shapiro–Wilk test. In order to determine between which points significant differences in parameter values occurred, the test was performed (a test of multiple comparisons of mean ranks for all samples tested). The parameters assumed statistically higher values at a given control-measurement point, in relation to values recorded at other points, if the median value determined from the dataset for this point was the largest. A significance level of α = 0.05 was assumed for all tests. Statistical analyses involved the use of STATISTICA version 12.5.

The research presents a technique in exploratory data analysis in multidimensional space. These methods do not take into account the class of the objects examined. Their goal is to express multidimensional observations using a small number of coordinates, maintaining specific relations between them as best as possible. The principal component analysis (PCA) finds the linear combinations of the original variables (mutually uncorrelated) that keep the maximum of the original variance of the data. Canonical correlation is an additional procedure for estimating the relationship between variables. In particular, this analysis allows studying the relationship between two sets of variables. Correspondence analysis is a descriptive and exploratory technique of analysis of bipartite and cross-tables, containing some measures characterizing the relationship between columns and rows. The obtained results provide information with similar properties as the results obtained in the case of factor analysis techniques and allow estimating the structure of these qualitative variables that create a data table. The purpose of using factor analytical techniques was to isolate these physicochemical parameters of water, which are related to land use and to reduce the number of variables to detect structures between variables and classify the studied variables into groups. The analysis uses a correlation matrix consisting of 196 samples for all physicochemical parameters. Multivariate analyses of PCA and CCA (canonical correspondence analysis) were performed using Canoco software for Windows (version 4.51).

### Water quality indices

The water quality index (WQI) calculated according to the adopted formula (Balan et al. 2012):

$$ WQI=\sum in=1\  Qi\  Wi/{\sum}_{i=1}^n Wi $$

where

*Qi*: Quality index for each *i* parameter
*Wi*: Calculated weight for each parameter
*n*: Number of parameters

The quality index (*Qi*) was enumerated using the following equation (Tripathy and Sahu 2005):

$$ Qi=\frac{\left( Vi- Vo\right)}{\left( Si- Vo\right)} $$

where

*Vi*: Measured value of the *i* parameter
*Si*: Standard allowable value of the *i* parameter
*V*_*o*_: Value of the *i* parameter in clean water

The index (*Wi*) was obtained by calculating the inverse proportionality for the recommended standards of water quality parameters:

$$ Wi=K/ Si $$

where

*K*: Constant proportional value for successive quality parameters expressed by the equation:

$$ K=1/\varSigma \left(1/ Si\right) $$

Metal index (*Mi*) was used to determine the overall trend of water quality. It describes the degree of water self-purification by computing the current trend of concentration of metals in water:

$$ MI=\sum i=1 nCi\ (MAC) $$

where

*Ci*: Concentration of individual metals
*MAC*: Maximum allowable concentration (quality status, to which high quality water should aspire)

### GIS technique

Area visualization involved the NTM (numerical terrain model) obtained from https://earthexplorer.usgs.gov/ from mission Routine ASTER Global Digital Elevation Model. The NMT spatial resolution was 25 m/px. Based on NMT, a height map and a relief of the analyzed area were made. The CORINE database of 2012 was used for general depiction of land use and land cover. The diversity of land cover and land use is shown precisely on the basis of the current ortho-photomap for the year 2015, with spatial accuracy of 0.25 m/px, obtained from the Polish institution responsible for collecting and sharing public spatial data—Geodesic and Cartographic Documentation Center. All GIS studies were carried out using QGIS 2.18 Las Palmas.

## Results

### Physicochemical indices

The average values of EC and concentration of chlorides and calcium at a short distance from the source of the watercourse (sampling site 1) decreased along the river. Concentrations of total dissolved substances, magnesium, potassium, nitrite nitrogen, phosphate phosphorus, iron, and manganese were also gradually decreasing from point 4 on the meandering section of the river, at the point located behind the sewage treatment plant and agricultural areas, to the last point (sampling site 7). The decreasing trend of values along the river was also observed in the case of the values of biochemical and chemical oxygen demand. Their values were reduced from the point located behind the sewage treatment plant (sampling site 3). The Kruskal–Wallis ANOVA test results showed statistically insignificant differences between the measurement/control points for temperature, total suspension, DO, chemical oxygen demand, EC, water pH, nitrate nitrogen, and manganese. Differences in values between points for the remaining 12 parameters were statistically significant (Table 1). In the first point, located in forest areas near the river source, there were statistically significant differences in the value of nine parameters compared to all the other points (Table 2). Concentrations of chlorides, sodium, and dissolved substances were higher at this point than those found at points located in the lower stretches of the watercourse (sampling sites 6 and 7). In addition, at this point as well, the concentration of ammonium nitrogen and the value of biochemical oxygen demand were higher than those found in point 6, and calcium concentrations than those found in sampling site 7. In one sampling site, there were lower concentrations of potassium, sulfates, and phosphate phosphorus in relation to those found in the middle stretch of the river. Potassium concentrations at the point located in the areas used for agriculture (sampling site 2), potassium and sulfates at the point behind the sewage treatment plant (sampling site 3), and also phosphate phosphorus in sampling sites 4 and 5 were higher than those found in sampling site 1. At the point located in the areas used for agriculture (sampling site 2), concentrations of chlorides, sodium, potassium, dissolved substances (sampling sites 6 and 7), and calcium (sampling site 7) were statistically significantly higher, compared to those recorded in the lower sections of the river. Concentrations of sodium, calcium, dissolved substances, and the value of biochemical oxygen demand in the middle course of the river at the point located after the treatment plant (sampling site 3) were also statistically significantly higher than the values found in the lower reaches of the river. Behind the water intake into anthropogenic facility, there were higher concentrations of phosphate phosphorus than recorded in the lower sections of the river. At the sampling site located along the meandering section of the river, before entering the water to the anthropogenic facility (sampling site 4), 9 parameters, out of 12 statistically significant ones, showed higher values in relation to the values of the same parameters in the lower sections of Skawa (Table 1).

**Table 1:** Descriptive statistics range of values with mean of the Skawa river water quality indices concentrations and comparison of analyzed water indices values between the sampling sites using Kruskal–Wallis test

Variables	Unit	Sampling sites							*p*
		1	2	3	4	5	6	7	
		Range value (average)							
Temperature	°C	− 0.1–10.8 (3.8)	− 0.1–12.3 (5.0)	− 0.1–11.7 (4.6)	− 0.1–11.4 (4.6)	− 0.1–11.6 (4.6)	− 0.1–12.7 (5.2)	− 0.1–13.3 (5.4)	0.995
Total suspended solids	mg dm^−3^	1.0–3.0 (2.0)	0.0–3.6 (1.3)	0.8–6.7 (2.8)	1.5–7.9 (3.1)	1.3–2.6 (1.9)	0.8–2.9 (1.6)	1.0–2.9 (1.6)	0.123
Saturated oxygen	%	38.0–136.0 (83.0)	94.0–131.0 (106.7)	46.0–131.0 (94.6)	77.0–123.0 (98.7)	84.0–133.0 (103.6)	84.0–128.0 (103.9)	75.0–138.0 (101.4)	0.566
Dissolved oxygen	mg O_2_ dm^−3^	3.9–17.3 (10.6)	10.6–16.3 (13.8)	5.0–16.7 (11.9)	9.4–15.9 (12.2)	9.3–16.3 (12.8)	9.3–15.6 (12.8)	8.3–14.4 (12.3)	0.666
BOD5	mg O_2_ dm^−3^	1.3–3.2 (2.3)	1.0–1.8 (1.3)	1.1–4.5 (2.5)	1.0–2.7 (1.6)	0.4–2.0 (1.3)	0.6–2.5 (1.1)	0.4–1.6 (1.0)	0.002
COD-Mn	mg O_2_ dm^−3^	2.4–9.5 (5.3)	1.5–6.0 (3.6)	1.8–8.9 (4.2)	2.7–6.4 (4.0)	2.4–6.0 (3.8)	1.8–5.0 (3.3)	0.5–6.5 (3.2)	0.353
Electrical conductivity	μS cm^−1^	402–1305 (636)	406–1186 (626)	403–1186 (617)	476–636 (554)	436–636 (511)	315–636 (430)	228–636 (395)	0.179
Total dissolved solids	mg dm^−3^	200–671 (345)	234–583 (326)	200–341 (262)	208–319 (273)	180–273 (228)	150–200 (180)	130–190 (146)	0.000
SO_4_	mg SO_4_ dm^−3^	12.0–23.2 (17.1)	17.5–27.3 (23.5)	22.6–36.4 (26.7)	21.0–31.2 (25.5)	20.9–31.0 (26.3)	19.2–27.8 (23.5)	18.8–28.4 (22.2)	0.005
Cl	mg Cl dm^−3^	51.5–339.6 (116.0)	31.1–181.6 (69.1)	21.0–62.2 (40.1)	23.4–53.7 (38.1)	23.6–49.6 (33.6)	13.5–23.6 (17.8)	5.8–21.0 (12.3)	0.000
Ca	mg Ca dm^−3^	38.5–107.2 (60.6)	43.7–92.5 (58.8)	43.6–94.6 (58.0)	44.4–92.2 (57.9)	42.4–76.6 (52.4)	36.1–51.1 (41.8)	31.9–42.7 (36.3)	0.000
Mg	mg Mg dm^−3^	7.5–18.3 (12.3)	7.5–18.0 (11.1)	7.4–19.1 (11.0)	7.8–19.3 (11.3)	7.6–17.1 (10.6)	6.3–11.4 (8.1)	6.2–9.5 (7.4)	0.008
Na	mg Na dm^−3^	33.8–162.7 (64.1)	20.8–107.7 (41.1)	16.5–38.7 (26.2)	17.5–30.3 (24.7)	16.9–27.3 (20.7)	11.4–14.9 (12.8)	6.2–12.9 (9.8)	0.000
K	mg K dm^−3^	1.0–2.8 (1.9)	4.2–6.8 (5.3)	2.8–6.8 (3.8)	2.9–5.7 (3.8)	2.5–4.3 (3.1)	2.4–3.1 (2.6)	1.5–2.6 (2.1)	0.000
pH in water	–	7.0–8.1 (7.6)	7.5–8.7 (8.2)	7.1–8.5 (7.9)	7.1–8.3 (7.8)	7.2–8.3 (7.9)	7.4–8.5 (8.0)	7.4–8.8 (8.0)	0.214
NH_4_	mg NH_4_-N dm^−3^	0–1.24 (0.32)	0–0.19 (0.08)	0.03–1.25 (0.25)	0–0.26 (0.16)	0–0.13 (0.06)	0–0.03 (0.01)	0–0.30 (0.05)	0.004
N-NO_3_	mg NO_3_-N dm^−3^	0.11–1.20 (0.42)	0.25–1.81 (1.14)	0.12–1.82 (0.90)	0.45–1.24 (1.01)	0.60–1.30 (1.03)	0.42–1.31 (1.04)	0.58–1.27 (1.02)	0.051
N-NO_2_	mg NO_2_-N dm^−3^	0–0.044 (0.006)	0–0.021 (0.014)	0–0.040(0.015)	0.010–0.029 (0.018)	0.008–0.026 (0.016)	0–0.014 (0.006)	0–0.010 (0.003)	0.002
P-PO_4_	mg PO_4_-P dm^−3^	0.010–0.065 (0.033)	0.029–0.095 (0.056)	0.030–0.345 (0.087)	0.056–0.178 (0.096)	0.062–0.133 (0.084)	0.025–0.038 (0.031)	0.013–0.033 (0.022)	0.000
Fe	mg Fe dm^−3^	0.06–0.82 (0.25)	0–0.29 (0.06)	0–0.43 (0.15)	0.29–1.03 (0.49)	0.12–0.68 (0.30)	0–0.36 (0.14)	0–0.24 (0.09)	0.000
Mn	mg Mn dm^−3^	0–0.30 (0.09)	0–0.06 (0.03)	0–0.15 (0.05)	0.03–0.20(0.10)	0–0.13 (0.07)	0–0.09 (0.04)	0–0.05 (0.03)	0.274

**Table 2:** Significance of differences of water indices between the individual measurement-control points (sampling sites) on the Skawa river—Dunn’s test (the differences are statistically significant at *p* < 0.05)

1	2	3	4	5	6	7	Sampling site
–	K	SO_4_; K	P-PO_4_; SO4; K	P-PO_4_; SO4	N-NH_4_; Cl; Na; BOD5; TDS	Cl; Na; Ca; TDS	1
			Fe		Cl; Na; K; TDS	Cl; Na; Ca; K; TDS	2
					BOD5	Na; Ca; BOD5;TDS	3
					P-PO_4_; N-NH_4_	P-PO_4_; N-NO_2_; Na; Ca; Mg; Fe; K; TDS;	4
					P-PO_4_	P-PO_4_	5
					–	–	6
					–	–	7

The analyzed water samples in terms of magnesium content ranged from 6.2 to 19.3 mg dm^−3^. The pH of the examined water was maintained during the research period at an even level of nearly 7.8. The highest concentration of ammonium nitrogen was observed at 1.25 mg dm^−3^. The concentration of ammonium nitrogen decreased along with the course of the river. The lowest concentrations of nitrate nitrogen were recorded at 0.11 mg dm^−3^. The phosphate phosphorus content was above 0.01 mg dm^−3^. The maximum calcium value in the watercourse studied was 107.2 mg dm^−3^. Due to the ease and rate of determining the EC of water, it is often used as a preliminary indicator of contamination and mineralization. The highest value of EC was 1305 μS cm^−1^ in the source cross section of Skawa. The content of oxygen dissolved in water ranged from up to 4.96 mg O_2_ dm^−3^. The highest concentration of biological oxygen demand was 4.5 mg O_2_ dm^−3^. The highest concentration of chemical oxygen demand in the studied watercourse reached the value of 9.5 mg O_2_ dm^−3^. The total dissolved substances along the course of the Skawa river ranged from 130 to 671 mg dm^−3^. Apart from the first sampling site, the chloride content did not exceed 339.6 mg dm^−3^ (Table 1).

**Fig. 3:**
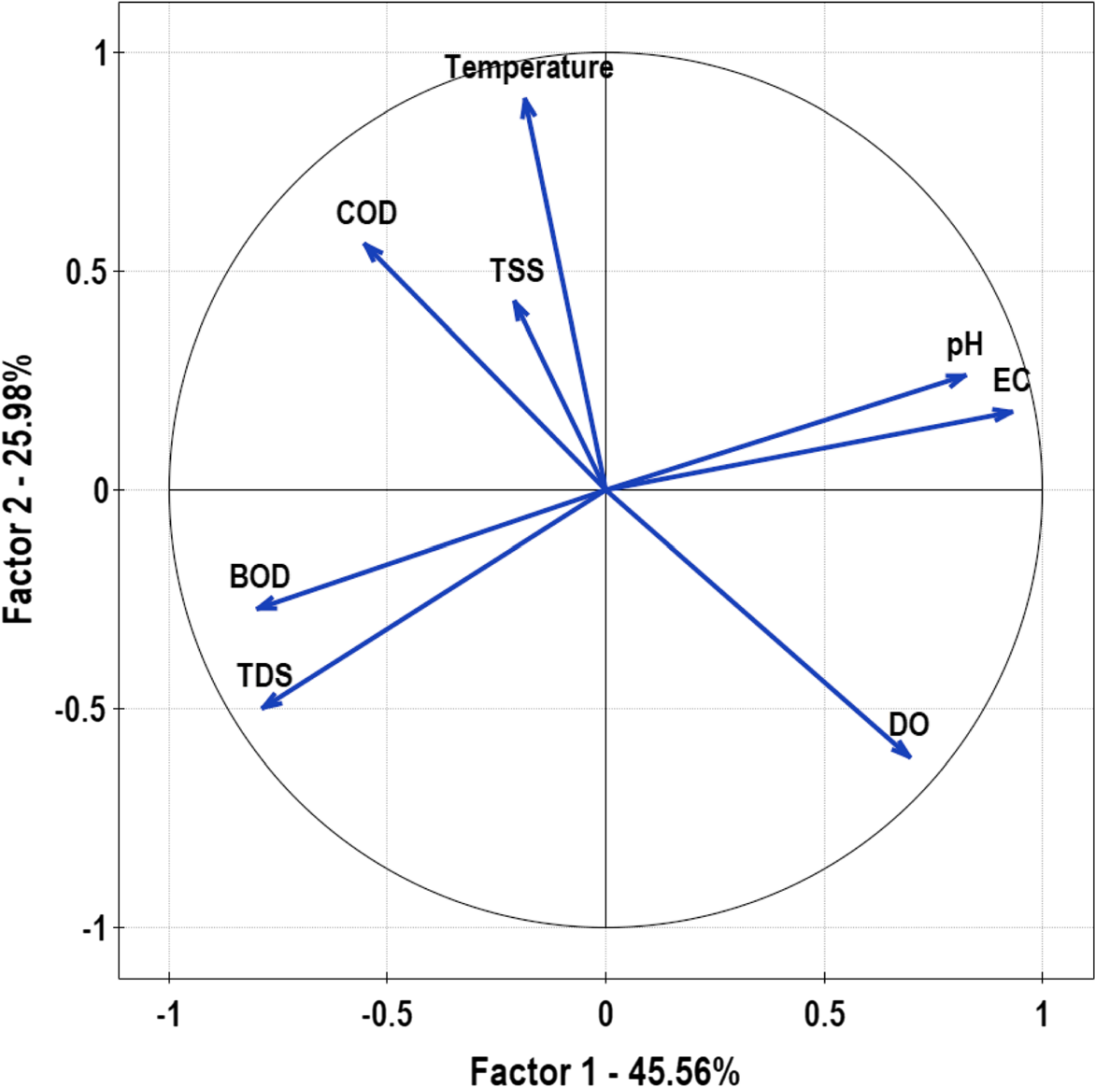
Multivariate PCA showing the most significant physicochemical parameters for the whole watershed. The analysis showed that the most influential variables were EC and pH. Statically significant differences were calculated using the Barllet test (*p* < 0.001). Correlations between variables and the main gradient were determined by the coefficient KMO = 0.453

**Fig. 4:**
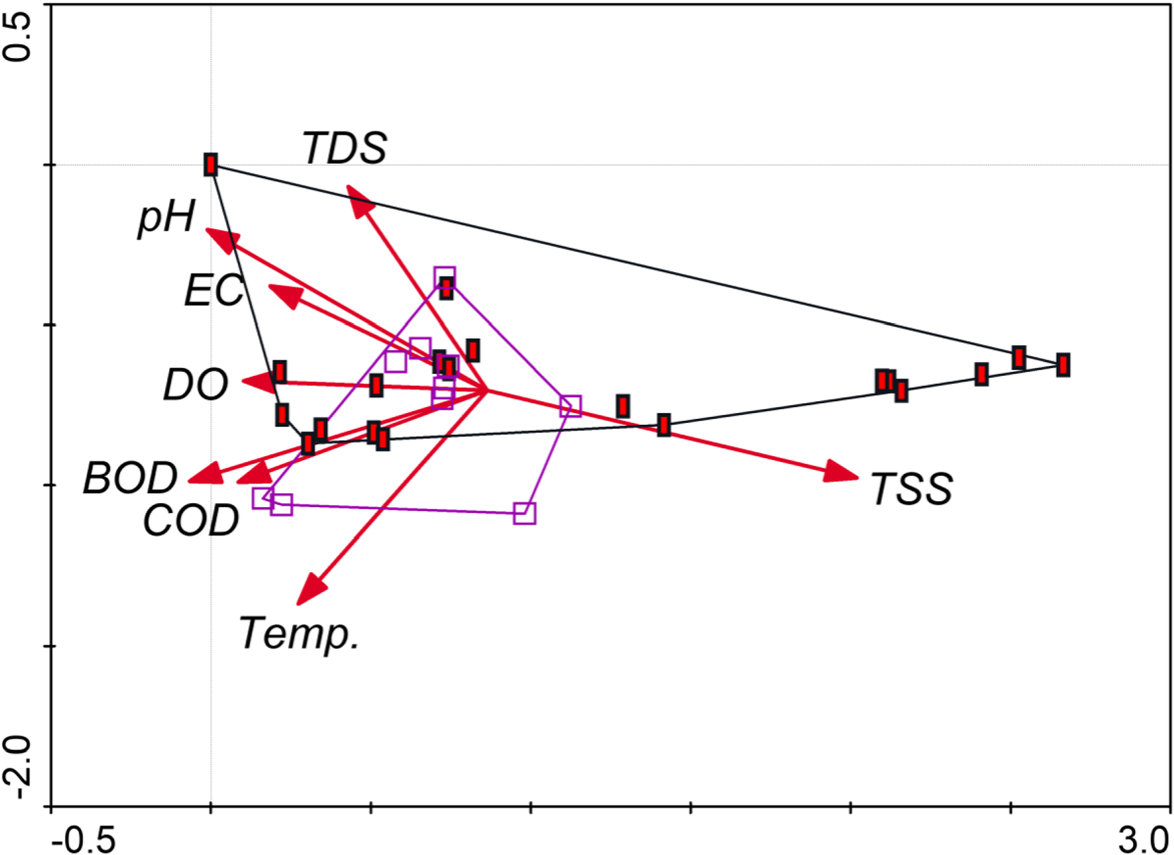
Multivariate CCA showing the types of land use that turned out to be statistically significant in the hydrochemical evaluation in the SAR spatial model. The solid rectangles illustrate non-irrigated land, and the open squares represent pastures. Vector arrows explain relationships between plots. TSS was positively correlated with land use, whereas temperature, COD, and to a lesser extent, BOD showed negative dependencies while assessing other physicochemical parameters. The first axis explained 54.6% of the variance. The second axis was responsible for 25.5% of the variance

WQI value for waters in flysch river reached the value of 45–68 for areas near the forest, 65–85 for agricultural areas, and 59–94 for the sampling site located near the quarry (Table 3). Results from the PCA showed the loading that each input variable (Table 4) contributes to the two principal components (Fig. 3). CCA was applied to the data to view the interrelation among the studied variables and their influence on land use. Land principally occupied by agriculture was positively correlated with total dissolved solids and total suspended solids (Fig. 4).

**Table 3:** WQI values for the most important land use types obtained from spatial autoregression model in the study area

Sampling sites	WQI		
	Non-irrigated arable land	Land principally occupied by agriculture	Pastures
1	66	45	70
2	81	85	67
3	68	79	69
4	80	76	68
5	74	77	72
6	73	68	73
7	67	94	59

**Table 4:** Loadings of environmental variables (eigenvectors) for PC’s axis made using PCA analysis determined the primary significance (validity) for the variables tested. The factor loadings of greatest importance have values above 0.80 and are italicized

Loadings factors	PC 1	PC 2	PC 3	PC 4	PC 5	PC 6
N-NO_3_	− 0.23	0.16	0.51	0.02	0.05	− 0.19
N-NO_2_	0.19	− 0.17	0.57	0.52	− 0.02	0.10
N-NH_4_	− 0.28	− 0.16	0.55	0.56	− 0.08	0.01
P-PO_4_	0.45	0.24	0.76	0.47	0.64	0.56
EC	*0.84*	*0.95*	0.52	− 0.32	− 0.02	0.06
DO	*0.87*	− 0.79	0.50	0.21	0.26	0.46
pH	*0.86*	*0.81*	0.59	− 0.35	− 0.01	− 0.2
TDS	*− 0.92*	*− 0.89*	*0.84*	− 0.01	0.06	0.01
Temperature	*− 0.80*	*− 0.89*	0.50	0.13	0.01	0.08
BOD5	− 0.79	*− 0.81*	*0.81*	0.002	0.21	0.04
COD	− 0.75	0.77	0.36	0.04	− 0.23	− 0.04
Eigenvalue	4.46	2.36	1.46	0.40	0.50	0.07
Explained variance (%)	45.56	25.98	18.40	5.06	3.00	2.00

### Application of the spatial water quality assessment model

The results of using the MESS (Matrix Exponential Spatial Specification) model showed that the increase in variables concerned only one type of use (Table 5); the effect it induced was smaller than that resulting from linear spatial autoregression. On the other hand, when the increase concerned two types of near-distance uses (Table 6), the impact of changes was greater than the estimated result of spatial autoregression. Our results indicate that the physicochemical parameters acting in relation to the types of use have a greater effect than in the absence of such relations. This was especially well reflected in connections with arable land, but is not reflected in the regression model. Both considered examples also point to relatively accurate results for the sandstone mine. The effect of changing the variable was not measured only using the MESS parameter. It is also influenced by the strength of connections between spatial units when interpreting parameters. Analogically to the MESS model, during the estimation of the SAR model, it turned out that only the linear trend was significant (Table 5). Both in the MESS model and in the SAR model, the parameters with the spatial trend and the explanatory variables were at a similar α-level (*p* < 0.05). The difference is in the parameters responsible for spatial autocorrelation. Therefore, in subsequent studies, we suggest taking into account the various spatial relationships between these parameters for the MESS model, and the *ρ-*parameter in the SAR model should be approximately equal to 1. Dependence of the autoregression parameter at 0.53 instead of the one obtained at 0.63. Adjusting the SAR model with the integrated MESS function is better than the SAR model; this means that in the case in question, the SAR model did not estimate spatial autocorrelation. Therefore, the improved SAR model described spatial relationship and the attributive data with more efficiently performance.

**Table 5:** Results of the SAR model with the MESS function for significant physicochemical parameters received while analyzing variance using the Kruskal–Wallis test. The response variable was the type of land use. The predictor variables were separated following the results of CCA analysis, selecting variables in terms of their significance and the strongest impact

Variable	Gamma	Coefficient	Standard error for gamma	Standard coefficient	Standard error	*t*	*p* value
N-NO_3_	3.93	1.04	0.56	1.30	0.02	1.44	3.09
N-NO_2_	2.04	2.09	0.78	1.09	0.32	− 2.94	0.02
P-PO_4_	5.01	1.94	0.89	2.02	0.82	3.45	0.43
Ca	6.36	0.94	− 0.09	− 0.09	0.01	− 1.95	0.09
Mg	3.57	0.43	0.02	0.81	1.03	− 2.75	0.70
Na	0.94	7.99	0.01	0.02	0.04	0.87	0.45
K	0.56	5.26	− 0.07	− 0.01	0.03	− 0.57	0.59
SO_4_	5.61	2.78	0.043	0.93	1.08	6.04	0.45
Cl^−^	3.78	1.45	0.35	0.01	0.30	7.67	4.56
EC	1.04	2.40	0.04	0.90	0.04	3.56	0.03
DO	2.45	0.36	0.76	1.94	0.05	3.05	0.004
COD	0.93	0.25	− 0.47	0.64	0.12	8.04	3.54
BOD	0.38	1.40	1.25	1.93	0.23	2.54	1.09
pH	1.67	3.75	0.92	1.85	1.90	4.02	1.92
TSS	4.78	2.30	0.45	0.45	3.66	1.57	0.002

**Table 6:** Results of the SAR model for land use

Land use	OLS coefficient	SAR coefficient	Standard coefficient	Standard error	*t*	*p* value
Constant	28.09	60.82	12.05	13.30	6.76	> 0.05
Discontinuous urban fabric	10.09	14.02	8.09	4.02	3.56	> 0.05
Industrial or commercial units	11.35	12.04	6.55	3.76	3.03	> 0.05
Mineral extraction sites	16.56	15.56	3.56	3.86	− 0.65	> 0.05
Non-irrigated arable land	9.56	14.46	5.78	0.32	2.01	< 0.05
Fruit trees and berry plantations	5.78	3.35	1.54	0.10	0.67	> 0.05
Pastures	2.67	1.19	0.57	− 2.81	− 0.56	< 0.05
Complex cultivation pattern	5.64	1.33	4.56	32.59	2.51	> 0.05
Land principally occupied by agriculture	14.67	12.99	3.87	− 4.45	− 3.13	< 0.05
Broad-leaved forest	6.56	6.71	4.56	4.56	4.56	> 0.05
Coniferous forest	10.78	9.45	5.56	− 0.45	0.79	> 0.05
Mixed forest	3.67	4.78	0.034	0.25	0.23	> 0.05
Transitional woodland-shrub	5.65	2.70	0.75	0.76	− 0.45	> 0.05

OLS (ordinary least squares) result: *r* = 0.547; *R*^2^ = 0.299; AICc = 12.554. Explained by predictor variables: *r* = 0.475; *R*^2^ = 0.565; AICc = 15.566. Total explained (predictor + space): *r* = 0.594; *R*^2^ = 0.534; AICc = 10.174

## Discussion

### Surface water quality indices

The factors that have the strongest impact on water quality include: soil type, slope steepness (Sheikh et al. 2014; Sungur et al. 2014), cultivated plant, and vegetation cover on soil in the embankment (Andersson et al. 2015; Teixeira and Marques 2016). Changes in nutrient concentration in sewage water are significantly affected by the distribution of agricultural land (Arienzo et al. 2012; Souza et al. 2012; Tasdighi et al. 2017). It should be emphasized that, regardless of the use of areas adjacent to the watercourse, the content of nitrates in the waters of the Skawa river decreased during the growing season. In summer and early autumn, the nitrate nitrogen content decreased, which is associated with the intense growth of plants and their greater nutritional needs (Ulén et al. 2012).

Cations (Ca^2+^, Mg^2+^), anions (NO_2_^−^, NO_3_^−^, SO_4_^2−^ and PO_4_^3−^), pH, TDS, EC, turbidity, fine clastic material in the form of suspended matter (general turbidity), are to be examined as per WHO standards (Shigut et al. 2017). Seasonal assessment of temperature, pH, chemical oxygen demand (COD), biochemical oxygen demand (BOD), heavy metals Fe, Mn, Ni, Cd, Cr, Co, Cu, Pb, and Zn as well as sulfates, nitrates and phosphates, is also needed for the recommended level of acceptability of drinking water pollution (Vincent-Akpu et al. 2015). DO is important for the assessment of water quality (Matta et al. 2017). According to Parmar and Keshari 2012, higher values of DO and BOD_5_ are associated with their high sensitivity to changes in water due to water engineering practices. It was noted that significant differences between BOD_5_ and COD_5_ are of anthropogenic origin during the assessment of seasonal fluctuations (Wałęga et al. 2018). The values of most parameters in the source part of Skawa (sampling sites 1 and 2) showed greater variability than at points located in the central (sampling sites 3, 4 and 5) and lower stretches of the river (sampling sites 6 and 7). A smaller variation in parameter values at these points may indicate better control of pollution entering the river and regulated water and sewage management. Higher diversification in the middle and lower stretches of Skawa was characterized only by magnesium, phosphate phosphorus – at the point located behind the wastewater treatment plant, slaughterhouse and tannery (sampling site 3) and the value of BOD (sampling site 6), as well as the pH value of water, COD and ammonium nitrogen concentration at the point behind the sandstone mine and mechanical and biological wastewater treatment plant (sampling site 7) (Table 1).

### Water quality assessment and sources of pollution

Small values of such hydrochemical indicator as WQI for reduction conditions and limited water exchange, while in other waters tested for oxidation conditions and intensive water exchange (Alobaidy et al. 2010; Balan et al. 2012; Yadav et al. 2015; Krishnan et al. 2016). Small values of the index in the examined waters suggest low agricultural development. High values of the indicator show active water exchange and good supply by infiltrating rainwater (Park et al. 2011), while small values (below 1.0), especially with low mineralization, indicate the possibility of surface water supply (Bu et al. 2014; Tiwari et al. 2015) or hydrodynamic stagnation (Banerjee and Srivastava 2009; Naubi et al. 2016). The WQI value also depends on the type of supply or type of rock (Tirone et al. 2010). This index for waters in flysch currents reached the value of 45–68 for areas near the forest, 65–85 for agricultural areas, 59–94 for the sampling site located near the quarry (Table 3). In the assessment of water quality, this value will be very useful when discussing the variables studied. Our results indicated a high degree of self-purification of water. High values for surface water accumulating on rocks may designate active ion exchange with infiltration waters from surface runoff as a result of the water erosion of soil (Jayawardane et al. 2011; Priess et al. 2015; Shi et al. 2016).

The physicochemical parameters of water quality can be used to illustrate general trends associated with the hydrogeochemical environment prevailing in the catchment (Sharma et al. 2012; Krishna Kumar et al. 2014; Zahra et al. 2014; Yan et al. 2015; Haritash et al. 2016), while water quality indices are used as hydrochemical standards for determining pollution sources (Trivedi 2010; Varol 2011; Xu et al. 2014; Effendi et al. 2015; Kumar et al. 2016). Also in the source part of the river, as compared to the remaining points, the highest mean values were shown for most indices characterizing aerobic conditions (sampling site 2), salinity (sampling site 1), the highest mean values of the acidification index (sampling site 2) and two biogenic ones - ammonium nitrogen (sampling site 1) and nitrate nitrogen (sampling site 2). The average values of other parameters analyzed from the group of indices characterizing the biogenic conditions - nitrite nitrogen and phosphate phosphorus and total suspended solids as well as iron and manganese, were the largest in the meandering middle section of the river, at the point located behind the sewage treatment plant, tannery and slaughterhouse (sampling site 4). The highest average water temperature was recorded at the point located behind the sandstone mine and the mechanical and biological wastewater treatment plant (sampling site 7). From the group of oxygen indices, only the average value of the BOD, and from the group of salinity indices, the average concentrations of sulfates were the highest in the middle stretch of the river (sampling site 3). In the source part of the river in forested areas (sampling site 1), the smallest mean content of DO in water was recorded, while the highest was recorded at the point located below, behind areas used for agriculture with a relatively dense development and an incomplete sewage system (sampling site 2) (Table 1).

### Impact of agricultural land on water quality

The characteristic of hydrochemical indices of surface water present in mountainous terrain has been presented. The use of GIS techniques allows determining the anthropogenic impact on water quality (Merem et al. 2011; Bora and Goswami 2014; Gernez et al. 2015; Sha and Ahmad 2015; Gholizadeh et al. 2016; Thapa et al. 2017a), spatial distribution of resources and water quality (Meng et al. 2015; Thapa et al. 2017b) and the demand for irrigation purposes (Misaghi et al. 2017). Seasonal fluctuations in such indices as concentration of DO and pH are, in turn, strongly correlated with the change in the structure of use (Bu et al. 2014; Bora and Goswami 2015). Fluctuations in the value of water quality parameters may be related to the spatial structure of land use (Hasani et al. 2015; Bora and Goswami 2016). In our results concentrations of phosphates, sulfates, magnesium and sodium cations as well as the level of EC did not present clear regularities. The structural-utility and production transformations have positively influenced the value of the flysch river, as well as the quality of waters in the analyzed mountainous catchment. In the vicinity of agricultural and forest areas, statistically significant differences occurred only between selected physicochemical properties (Table 2), however in the spatial layout taking into account the entire area, dependency of the catchment’s use was identified (Table 5). The spatial dependencies between land use showed diversity within the whole catchment (Tu and Xia 2008; Phung et al. 2015). Studies of relations in a spatial system are important due to the recognition of spatio-temporal patterns and the associated sources of pollution (Su et al. 2011; Scheili et al. 2015; Thapa et al. 2017c). At the research sampling site located close to the sandstone mine, they decrease with the occurrence of a greater concentration of TSS. We did not observe statistically significant differences between the majority of the indices examined. Also, the analysis of spatial autoregression did not show the dependency between the dependent variable (the variant of use) and the independent variables (predictors), like physicochemical parameters. In contrast to these results, an analysis of spatial autoregression with the MESS equation showed the impact of only total suspended solids, nitrite nitrogen, DO, and EC on water quality. The physicochemical indices obtained may be proposed for the selection of factors useful in the hydrochemical assessment (Liu et al. 2010; Thapa et al. 2017d) or modeling relationships between catchment attributes and water quality using spatial regression (Yang et al. 2017).

### Solutions for practical purposes and water protection

Erosion of mountain soils, is the subject of research related to water engineering. Depending on the volume of flow, the intensity of transport of suspended sediment flowing into the stream is a major issue, because the assessment of its intensity is important in agriculture (Halecki et al. 2018a, b, c). The development of variability of the main sources of transported weathered material is important in sedimentology, and the intensity of suspended sediment, activated by surface run-offs and its delivery during high-intensity rainfall in the assessment of surface water erosion of soil (Halecki et al. 2018a). The obtained research results indicate that the land use shapes the structure of waterside water quality, as well as affects the condition of watercourses, especially in terms of their content of nitrates, phosphates and sulfates (Akan et al. 2012; Wallender and Tanji 2012) and sodium, potassium, calcium, sodium and magnesium cations (Purandara et al. 2012). The most Ca, Mg, Na, K, Fe, N-NO_2_, Cl^−^ and SO_4_ were found in waters flowing through a field complex in the spring (Padmalal et al. 2012). According to Bu et al. (2017), this process is associated with greater biological sorption of minerals by plants grown in summer, and smaller by plants in the spring.

The highest values of the MI index 2.55 (Ca^2+^) and 2.32 (Mg^2+^), are shown by mineralized waters and water near forests, respectively, with dispersed household buildings, providing for limited water exchange areas. However, the lowest values of MI, 1.29 (Ca^2+^) and 1.47 (Mg^2+^), indicate self-purification of water. A reverse dependency was found for the content of Na^+^ and K^+^; this may be caused by the inability to use all of these components by plants growing on the surrounding hills. A number of primary ions took the following sequence Ca^2+^ > Cl^−^ > Na^+^ > SO_4_^2−^ > Mg^2^ > K^+^ > Fe > Mn trend.

The water quality assessed using the multivariate statistical analysis indicated sources of pollution (Varol et al. 2012; Velleman and Welsch 2012; Ogwueleka 2015). In our study, the multivariate analysis of PCA showed the general trend and relations between pH, EC, DO, total dissolved sediments, BOD, COD, temperature, and total suspended solids (Fig. 3). The results contained in this article have shown that TDS, TSS, pH, temperature, COD, BOD, DO, and EC in the water were identified as the most influential parameters affecting non-irrigated arable lands and pastures (Fig. 4).

## Conclusion

The conducted studies have demonstrated the effectiveness of the MESS model as a tool for spatial analysis of physicochemical indices. The results of parameters characterized by spatial autocorrelation should take into account the MESS model. In the water quality tests, the fit of the model was weaker than for the SAR model. We, therefore, recommend caution when using it. However, our results indicated that the ease of estimating a MESS model may be of great importance when more data is being analyzed. In the MESS model, the *ρ-*parameter reflects the strength of spatial autocorrelation between the neighbors of the first order of the explained variable. The spatial autocovariance function, which also reflects the relationships of higher orders, takes the exponential form, unlike the SAR model, which decreases geometrically. For the estimation of the spatial model, it is worth simplifying the examined parameters, e.g. applying the highest likelihood method, since when considering the parameters of water quality and neighborhood impact of different types of use, the interpretation will be closer to the actual results, not only generated by the model with a specific pattern. Analysis of indices for the sampling site 1, which was located at the beginning of the built-up area, showed the highest average, which could be caused by the lack of regulated water and sewage management of a housing estate located near the sampling area. Distributed buildings and agricultural land had an impact on EC and total suspended solids. Smaller differentiation between parameter values in sampling sites 6 and 7 located in the residential area may indicate enhanced control of pollution entering the river and regulated water and sewage management in the area. The quarry - did not affect the water quality, probably due to the sophisticated protection of the excavation through green buffer belts near the river bed. The unsatisfactory quality of water in the Skawa river was caused by excessive values for the concentration of nutrients. Higher concentrations of ammonium nitrogen and nitrate nitrogen were recorded in winter months, probably due to the inhibitory effect of biological and biochemical processes through the nitrification and assimilation of nitrate nitrogen. Water from the Skawa river may be intended for human consumption, provided that it is subjected to highly efficient physical and chemical treatment. However, action should be taken to limit the concentration of phosphate phosphorus. Based on the control of surface waters, it is possible to determine the primary impurities found in water, and thus the sources of these pollutants, which, if properly identified, will allow developing effective methods for improving water quality and the means of protection in this area.

## Electronic supplementary material

ESM 1 (DOCX 13 kb)
