# Supplementary material for: Evaluating the applicability of MESS (matrix exponential spatial specification) model to assess water quality using GIS technique in agricultural mountain catchment (Western Carpathian)
Source: Environ Monit Assess. 2018 Dec 21;191(1):26. doi: 10.1007/s10661-018-7137-x (PMC6302058; doi:10.1007/s10661-018-7137-x)
Supplement: Supplementary file 1 — (DOCX 13 kb) [file 10661_2018_7137_MOESM1_ESM.docx]

Table X. Factor loadings of studied land use calculated by Canonical Correspondence Analysis.

| Predictors/Land-use | FC 1 | FC 2 | FC 3 | FC 4 |
| --- | --- | --- | --- | --- |
| Constant | 0.52 | 0.49 | 0.32 | 0.04 |
| Discontinuous urban fabric | 0.69 | 0.62 | 0.59 | 0.21 |
| Industrial or commercial units | 0.54 | 0.59 | 0.45 | 0.71 |
| Mineral extraction sites | 0.48 | 0.45 | **0.82** | 0.40 |
| Non-irrigated arable land | **0.85** | **0.96** | 0.23 | 0.62 |
| Fruit trees and berry plantations | 0.40 | 0.34 | 0.19 | 0.78 |
| Pastures | **0.87** | **0.90** | **0.94** | 0.35 |
| Complex cultivation pattern | 0.28 | 0.60 | **0.91** | 0.57 |
| Land principally occupied by agriculture | 0.46 | 0.49 | 0.68 | 0.34 |
| Broad-leaved forest | 0.14 | 0.57 | 0.54 | 0.36 |
| Coniferous forest | 0.18 | 0.48 | 0.27 | 0.16 |
| Mixed forest | 0.52 | 0.49 | 0.18 | 0.07 |
| Transitional woodland-shrub | 0.30 | 0.39 | **0.93** | 0.39 |

Indicators were ranked in ascending or descending order. The most important eigenvalues (>0.8) were bolded. FC means factor component
